# Prediction of changes due to mandibular autorotation following miniplate-anchored intrusion of maxillary posterior teeth in open bite cases

**DOI:** 10.1186/s40510-018-0213-5

**Published:** 2018-05-14

**Authors:** Hassan E. Kassem, Eiman S. Marzouk

**Affiliations:** Department of Orthodontics, Faculty of Dentistry, Alexandria University, Alexandria, Egypt

**Keywords:** Intrusion, Skeletal anchorage, Miniplates, Autorotation, Prediction, Regression

## Abstract

**Background:**

Prediction of the treatment outcome of various orthodontic procedures is an essential part of treatment planning. Using skeletal anchorage for intrusion of posterior teeth is a relatively novel procedure for the treatment of anterior open bite in long-faced subjects.

**Methods:**

Data were analyzed from lateral cephalometric radiographs of a cohort of 28 open bite adult subjects treated with intrusion of the maxillary posterior segment with zygomatic miniplate anchorage. Mean ratios and regression equations were calculated for selected variables before and after intrusion.

**Results:**

Relative to molar intrusion, there was approximately 100% vertical change of the hard and soft tissue mention and 80% horizontal change of the hard and soft tissue pogonion. The overbite deepened two folds with 60% increase in overjet. The lower lip moved forward about 80% of the molar intrusion. Hard tissue pogonion and mention showed the strongest correlations with molar intrusion. There was a general agreement between regression equations and mean ratios at 3 mm molar intrusion.

**Conclusions:**

This study attempted to provide the clinician with a tool to predict the changes in key treatment variables following skeletally anchored maxillary molar intrusion and autorotation of the mandible.

## Background

The visualization of the treatment outcome of orthodontic therapy is an indispensable tool in the orthodontist armamentarium [1]. Prediction of the change in the orofacial complex has been given a lot of attention in the orthodontic literature. Orthodontists have been interested in predicting the changes due to growth [2, 3]. A lot of emphasis has been given to the prediction of changes in the soft tissues of the face which brought about orthognathic surgery [4, 5]. Prediction of the soft tissues following orthodontic tooth movement was reported particularly in lip response to premolar extraction and anterior retraction [6].

The advent of skeletal anchorage opened the door to treating many skeletal problems where orthognathic surgery has been classically the treatment of choice. Skeletal open bite and the long face syndrome are clear examples [7]. The classical orthognathic surgery involves Le Fort maxillary impaction with or without mandibular surgery. Most of the favorable effects were induced by the autorotation of the mandible, namely reduction of the skeletal and soft tissue facial heights, increase in the projection of hard and soft tissue chin points, reduction of the overjet, and increase of the overbite. In addition, the lower lip position changed with the autorotation of the mandible.

To serve the purpose of cephalometric prediction tracing, several authors attempted to relate the change in soft tissue projection to the degree of mandibular autorotation. Proffit [8] estimated that both the lower lip and soft tissue chin rotate at a ratio of 1:1 with the rotation of the mandible. Soft tissue chin point rotated at a 1:1 ratio with the hard tissue chin point on the same arc [9]. Similarly, Lee et al. [10] reported a 1:1 ratio between the mandibular soft tissue and hard tissue landmarks secondary to autorotation of the mandible following maxillary impaction. On the other hand, Mansour et al. [11] found that the soft tissue chin point and the mandibular sulcus point moved horizontally at a ratio of 0.86 (*r* = 0.92) and 0.91 (*r* = 0.91) relative to the corresponding hard tissue landmarks. Vertically, the soft tissue menton was displaced superiorly at a ratio of 1.2 relative to hard tissue menton (*r* = 0.79). Van Butsele et al. [12] reported that the lower lip moved 80% of the displacement of the menton by autorotation of the mandible.

Several authors attempted to correlate the changes brought about by maxillary impaction and mandibular autorotation to the amount of maxillary impaction. Bell et al. [13] described a 1:1 ratio of vertical and sagittal hard tissue chin movement relative to maxillary impaction. Similarly, Fish et al. [14] described the same vertical ratio; however, they reported 70% advancement of the mandible relative to the amount of maxillary impaction. The chin point was found to move forward 4.8 mm for 5.4 mm superior positioning measured at the occlusal point of the maxillary first molar (calculated ratio = 1:0.88; *r* = 0.789) [15]. This approach appears to be more clinically relevant since the amount of superior movement of the maxillary posterior teeth by either orthognathic surgery or orthodontic tooth movement is dictated by the treatment plan tailored to each individual case.

Similarly, Steinhäuser et al. [16] reported the ratios of vertical displacement of skeletal and soft tissue points to the superior movement of the posterior nasal spine to be 60.9% at menton (Me), pogonion ( Pg) 69.8%, lower incisor 84.4%, soft tissue menton (Me´) 60.4%, soft tissue pogonion (Pg´) 49%, and lower lip by 72.4%. Horizontal ratios were: Me 79.7%, Pg 78.6%, and lower lip 23.4%. Moreover, they reported a difference in response with the different types of maxillary impaction. The advancement of the pogonion was 100% in parallel impaction group, 50% in posterior impaction with additional anterior subsidence group and 80% in the exclusive posterior impaction group.

Before the introduction of miniplates and miniscrews, the treatment options for adult open bite patients were either orthognathic surgery involving at least maxillary Le Fort I impaction or orthodontic camouflage by extruding the anterior teeth. Intrusion of the posterior teeth anchored by miniplates and miniscrews was an attractive alternative that can be considered a nonsurgical maxillary impaction [17]. Furthermore, skeletal anchorage added the possibility of intruding mandibular posterior teeth to maximize the treatment benefit [18].

Several studies have reported on the findings of skeletally anchored maxillary posterior teeth intrusion [19–37]. However, few studies [30–32, 37] have reported soft tissue changes associated with posterior teeth intrusion (Table 1).

**Table 1:** Selected variables from studies of molar intrusion

	Intrusion	Pg	Pg′	N-Me/LAFH	Me′	Lower lip	Overjet	Overbite
Sherwood et al. [19] ^a^	− 1.99 ^g^			− 2.62 ^g^				3.62 ^g^
Sugawara et al. [18] ^a^	− 1.7 (0.91)			− 1.5 ^g^			− 1.3 ^g^	4.9 ^g^
Erverdi et al. [20] ^a^	− 2.6 (1.39)						− 2.0 (2.53)	3.7 (2.4)
Erverdi et al. [23] ^b^	− 3.6 (1.4)			− 2.9 (1.3)			− 1.4 (1.5)	5.1 (2.0)
Kuroda et al. [24] ^a^	− 2.3 (2.0)			− 3.6 (1.8)			3.6 (2.4)	6.8 (1.7)
Xun et al. [25] ^b^	− 1.8 (0.7)	2.5 (2.6) ^c^		− 1.6 (0.9)			−2.0 (2.2)	4.2 (0.9)
Lee and Park [26] ^a^	− 2.2 (1.7)	2.17 (2.47)		− 2.63 (1.96)				5.47 (1.86)
Akay et al. [27] ^b^	− 3.4 ^g^			− 3.7 ^g^				4.8 ^g^
Baek et al. [28] ^a^	− 2.39 (1.76)	2.4 (2.32)		− 2.53 (1.9)				5.56 (1.94)
Buschang et al. [29] ^a^	N/A	2.4 (2.3)	N/A	N/A	N/A	N/A	N/A	N/A
Deguchi et al. [30] ^a^	− 2.3 (1.3)			− 2.6 (2.5)		− 3.1 (2.7) ^d^	− 3.0 (2.9)	6.2 (1.7)
Akan et al. [31] ^b^	− 3.37 (1.21)			− 4.16 (1.71)		− 0.42 (1.17) ^e^	− 1.68 (2.0)	4.79 (1.36)
Foot et al. [32] ^b^	− 2.9 (0.8)			− 0.9 (1.1)			− 0.1 (1.2)	3.0 (1.5)
Scheffler et al. [33] ^b^	− 2.3 (1.4)			− 1.6 (2.2)				2.2 (1.6)
Hart et al. [34] ^b^	− 2.3 (0.06)			− 1.5 (0.03)			− 1.1 (1.4)	3.8 (0.94)
Marzouk et al. [35] ^b^	− 3.1 (0.74)						− 1.7 (0.82)	6.55 (1.83)
Marzouk and Kassem [36] ^a^	− 3.04 (0.79)	2.45 (0.05)	N/A	− 3.57 (1.15)	N/A	N/A	− 3.39 (2.04)	6.93 (1.99)
Marzouk and Kassem [37]^a^	− 3.04 (0.79)	N/A	2.43 (0.47)	N/A	− 3.12 (0.58)	− 1.15 (0.22) ^d^ − 1.23 (0.05) ^e^ 1.78 (0.74)^f^	N/A	N/A

^a^Measurements taken post-treatment and extractions were involved

^b^Measurements taken post-intrusion

^c^Measured at point B

^d^Measured to Sn-Pg′

^e^Measured to E-line

^f^Measured to true vertical

^g^S.D. not reported

We have previously reported on the skeletal, dental, and soft tissue effects following maxillary posterior intrusion using zygomatic miniplates [35–37]. The objective of this paper is to present regression models that help provide more accurate prediction of the effect of molar intrusion on several skeletal, dental, and soft tissue parameters important for the clinician.

## Methods

The data was derived from the sample previously reported by the authors [36, 37]. The subjects recruited were 28 young adults (range = 19–28 years) presenting with 3–8-mm anterior open bite and posterior vertical maxillary dentoalveolar excess as determined by initial cephalograms. Ethical approval was obtained from the Ethics Committee of the Faculty of Dentistry, Alexandria University, Egypt. Each subject signed an informed consent for the participation in the study.

The detailed protocol used for the maxillary posterior segment intrusion was previously described [35]. The maxillary posterior segments were leveled with sectional wires from the first premolar to the second permanent molar. After reaching 0.019 × 0.025 in stainless steel wire segment, a double transpalatal arch was fitted. Under local analgesia, titanium I-shaped miniplate (Gebrüder Martin GmbH & Co. KG, Tuttlingen, Germany) was screwed to the zygomatic buttress on each side. The lower end of the miniplate extended through the incision into the oral cavity and the terminal eyelet were modified into a hook. A NiTi coil spring (GAC, Bohemia, NY, USA) extended from the hook to the maxillary first molar, applied 450 g of intrusive force per side. The coil spring was replaced by a 0.012-in stainless steel ligature when the overbite reached 1 to 2 mm.

**Fig. 1:**
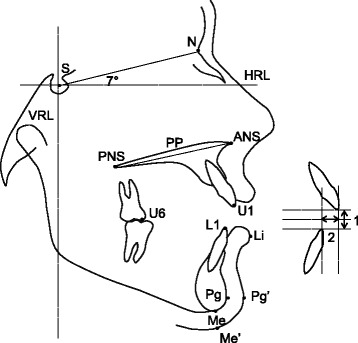
Landmarks and reference planes. S, sella; N, nasion; ANS, anterior nasal spine; PNS, posterior nasal spine; U6, mesial cusp tip of maxillary first molar; U1, incisal edge of maxillary incisor; L1, incisal edge of mandibular incisor; Li, labrale inferius the most anterior point of lower lip; Pg, hard tissue pogonion; Pg′, soft tissue pogonion; Me, hard tissue menton; Me′, soft tissue menton; HRL, horizontal reference line; VRL, vertical reference line; PP, palatal plane; 1, overbite measured along VRL; 2, overjet measured along HRL

Data in this report were collected from lateral cephalometric radiographs taken before intrusion (following the leveling and alignment) and after maxillary posterior teeth intrusion. The radiographs were taken with the teeth in occlusion and lips at repose. All radiographs were traced by hand, and landmarks were identified by one observer on standard acetate paper with a sharp pencil [38]. Landmarks relevant to this report are shown in Fig. 1.

Measurements were taken using a digital caliper to the nearest 0.05 mm. Magnification was compensated for using the ruler grid of the radiograph. The horizontal reference line (HRL) was constructed through S point at 7° to SN. A line perpendicular to HRL passing through S represented the vertical reference line (VRL) [39].

### Statistical analysis

The intra-rater and inter-rater reliabilities were previously calculated [36, 37]. Paired *t* tests showed no statistically significant differences between pairs of measurements. Same examiner intraclass correlation coefficients were greater than 0.90 for hard tissue measurements and 0.84 for soft tissue measurements. Intraclass correlation coefficients between examiners were greater than 0.87 and 0.79 for hard and soft tissue measurement, respectively. Ratios between pairs of measurements were calculated. The data were verified for normality of the measurements using histograms and Shapiro-Wilk tests. Scatter plots were used to confirm linearity and homoscedasticity. Paired *t* tests were used to compare pre-intrusion and post-intrusion variables. Pearson product moment correlation tests were performed to calculate regression coefficients and derive regression equations. The statistical analysis was done using the Statistical Package for the Social Science (SPSS, Version 20). Significance level was set at *P* ≤ 0.05 for paired *t* comparisons. Bonferroni correction was used to avoid type I error with multiple correlation testing; hence, significance level was set at *P* ≤ 0.01.

## Results

Table 2 shows selected variables before and after maxillary posterior teeth intrusion where the selected variable showed statistically significant differences.

**Table 2:** Comparison of selected variables before and after maxillary posterior teeth intrusion

	Before intrusion		After intrusion		Difference		
Variable (mm)	Mean	S.D.	Mean	S.D.	Mean	S.D.	*P* ^a^
U6-PP	28.31	2.48	25.27	2.23	− 3.04	0.79	^**^
Pg-VRL	44.87	2.39	47.29	2.12	2.42	0.19	^**^
Me-HRL	106.85	2.51	103.60	2.50	− 3.25	0.77	^**^
Pg′-VRL	73.72	1.32	76.18	1.08	2.46	0.28	^**^
Me′-HRL	114.35	1.29	111.18	1.53	− 3.17	0.42	^**^
Overbite	− 4.86	1.69	1.32	0.85	6.18	1.35	^**^
Overjet	5.71	1.38	3.84	1.56	− 1.87	0.44	^*^
Li-VRL	80.69	1.45	83.22	1.14	2.53	0.23	^**^

^a^Paired *t* test

*P ≤ 0.01; **P ≤ 0.001

Mean ratios between the amount of intrusion measured at the maxillary first molar and selected variables are reported in Table 3. The hard tissue chin point and the soft tissue chin points moved forward 79 and 80%, respectively, of the distance the maxillary first molar was intruded. The facial height at Me and Me′ decreased at approximately 1:1 of the maxillary first molar intrusion. The overbite was found to deepen two fold, whereas the overjet was reduced by 61% of the maxillary molar movement. The lower lip moved horizontally 83% of the amount of intrusion.

**Table 3:** Mean ratios between mean molar intrusion and selected variables

			Mean ratio
U6-PP	:	Pg-VRL	− 1.00:0.79
		Me-HRL	− 1.00:− 1.06
		Pg′-VRL	− 1.00:0.80
		Me′-HRL	− 1.00:− 1.04
		Overbite	− 1.00:2.03
		Overjet	− 1.00:− 0.61
		Li-VRL	− 1.00:0.83

Linear regression showed that upper molar intrusion was a significant predictor for all the selected variables (Table 4). Regression equations explained more than 50% of the variation of Pg-VRL, Me-HRL, overbite, overjet, and Me′-HRL. Less than 30% in the variability of Pg′-VRL and labrale inferius (Li)-VRL could be explained by the regression equations. Using the obtained regression equations, a typical 3-mm molar intrusion will result in 2.36 and 2.48 mm forward movement of Pg (*r* = − 0.88, *P* ≤ 0.001) and Pg′ (*r* = − 0.4, *P* ≤ 0.01), respectively, − 3.1-mm upward movement of both Me (*r* = 0.91, *P* ≤ 0.001) and Me′ (*r* = 0.77, *P* ≤ 0.001), 6.39 mm increase of the overbite (*r* = − 0.73, *P* ≤ 0.001), and 1.81-mm reduction in the overjet (*r* = 0.72, *P* ≤ 0.001). Prediction equation for the lower lip showed 2.55-mm forward movement for 3-mm intrusion (*r* = − 0.51, *P* ≤ 0.01).

**Table 4:** Regression between upper molar intrusion and selected variables

	Regression equation	*r* ^*2*^	*r* ^a^	*P*
Pg-VRL	1.79 + − 0.19 U6-PP	0.79	− 0.88	**
Me-HRL	− 0.63 + 0.83 U6-PP	0.82	0.91	**
Pg′-VRL	2.18 + − 0.10 U6-PP	0.16	− 0.40	*
Me′-HRL	− 1.96 + 0.38 U6-PP	0.59	0.77	**
Overbite	2.88 + − 1.17 U6-PP	0.53	− 0.73	**
Overjet	− 0.67 + 0.38 U6-PP	0.51	0.72	**
Li-VRL	2.13 + − 0.14 U6-PP	0.27	− 0.51	*

^a^Pearson moment correlation coefficient

*P ≤ 0.01; **P ≤ 0.001

**Fig. 2:**
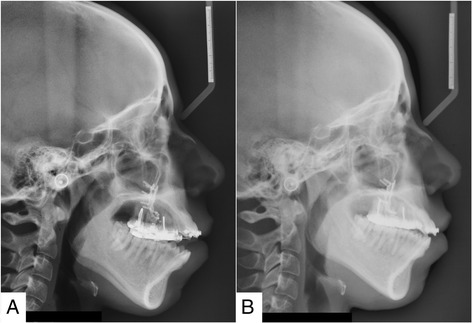
Lateral cephalometric radiographs of a subject: **a** before and **b** after maxillary posterior teeth intrusion

Table 5 shows a comparison between prediction of the selected variables according to calculated mean ratios and regression equations for 3 mm of molar intrusion. Representative lateral cephalometric radiographs before and after intrusion are shown in Fig. 2.

**Table 5:** Prediction of selected variables according to mean ratios and regression equations for 3 mm of molar intrusion

	Mean ratio	Regression
Pg-VRL	2.37	2.36
Me-HRL	− 3.18	− 3.12
Pg′-VRL	2.40	2.48
Me′-HRL	− 3.12	− 3.10
Overbite	6.09	6.39
Overjet	− 1.83	− 1.81
Li-VRL	2.49	2.55

## Discussion

Prediction of outcomes of various orthodontic procedures is important for treatment planning. The use of skeletal anchorage for molar intrusion in skeletal open bite patients allowed the orthodontists to yield orthopedic changes in adults using mandibular autorotation. In the current study, data from before and after intrusion were used to provide the clinician with tools to predict changes following intrusion of maxillary posterior teeth.

The authors chose the landmarks most likely to affect the clinician treatment decision following maxillary molar intrusion, namely the horizontal position of the chin, the face height, the overbite and the overjet, and the lower lip position.

In the present study, the hard tissue pogonion was found to move forward at a ratio of 1:0.79 to the maxillary molar intrusion. This is close to the findings of Lee and Park [26] who reported a ratio of 1:0.9. Xun et al. [25] reported a ratio of 1:1.3 for the forward movement of the point B. This modest difference between the two points can be explained that they do not necessarily lie on the same arc of rotation. Bell et al. [13] described a 1:1 ratio of vertical and sagittal hard tissue chin movement relative to maxillary impaction. On the other hand, Fish et al. [14] described reported 70% advancement of the mandible relative to the amount of maxillary impaction, which approximates the ratio obtained in the present study. Wang et al. [15] reported a strong correlation between the amount of maxillary superior positioning and the displacement of the chin following maxillary impaction and mandibular autorotation (*r* = 0.79) at a ratio of 1:0.88, which are similar to the findings of the present study. Moreover, Steinhäuser et al. [16] found that pogonion moved forward 78.6% of the distance of maxillary impaction measured at the posterior nasal spine. When measuring the change at pogonion in exclusive posterior impaction subjects, which resembles the effects of maxillary posterior segment intrusion, the forward advancement at pogonion was found to be 80% of the amount of maxillary impaction, which is in accordance with the current results.

The reduction of the facial height reported in the study (1:1) was found to agree with several authors [18, 26–28, 30]. Some studies reported lower [23, 25, 33, 34] and higher ratios [19, 24, 31]. The differences did not deviate largely from the present report; however, it can be explained by the different methods of measuring the facial height used in the different studies. In addition, some studies reported measurements post-treatment that may have involved extractions that may have altered the facial height [18, 19, 24, 26, 28, 30]. In samples of orthognathic surgery subjects treated with maxillary impaction, Bell et al. [13] and Fish et al. [14] reported 1:1 ratio of vertical displacement of the hard tissue chin relative to the amount of maxillary impaction following mandibular autorotation. Steinhäuser et al. [16] found the vertical displacement at Me was 60.9% of the amount of maxillary impaction measured at PNS. This may be attributed to the different methods used for maxillary impaction, where they reported different displacements at Pogonion depending on whether the impaction was parallel, posterior, or posterior with anterior lowering.

The soft tissue pogonion was found to move at the same ratio as the hard tissue pogonion. Similarly, soft tissue menton showed the same ratio of change as hard tissue menton. Schendel et al. [9] reported that the soft tissue chin point rotated at a 1:1 ratio with the hard tissue chin point on the same arc following mandibular autorotation due to maxillary impaction surgery. In addition, Lee et al. [10] reported a 1:1 ratio between the mandibular soft tissue and hard tissue landmarks secondary to autorotation of the mandible following maxillary impaction. On the other hand, Mansour et al. [11] reported that the soft tissue chin point moved horizontally at a ratio of 0.86 relative to the corresponding hard tissue chin, whereas the soft tissue menton displaced superiorly at a ratio of 1.2 relative to hard tissue menton. The explanation stated by the authors was the stretching of the soft tissue following the upward and forward displacement of the mandible after maxillary impaction surgery. Steinhäuser et al. [16] found an equal ratio for the vertical displacement of Me and Me′ (60.9 and 60.4%, respectively); the ratios, however, are smaller compared to the present study which may be attributed to the cumulative effect of the different methods of maxillary impaction used in their sample.

Comparing ratios of change of dental measurement will be limited to the few studies reporting values following intrusion [23, 25, 27, 31–34]. In the present study, the ratio of overbite correction to the amount of molar intrusion was approximately 1:2. This agrees with one of the prosthodontic tenets that each millimeter of molar intrusion yields a 2 to 3 mm closure of the anterior bite [40]. Similar ratios were reported by Xun et al. [25] and Hart et al. [34]. Smaller ratios were found by several authors [23, 27, 31–33]. This may be attributed to the compensatory eruption of mandibular molars during maxillary molar intrusion which was reported in other studies [18]. In the present study, a strict protocol was followed to avoid this effect [36].

The overjet was found to reduce by 60% of the amount of molar intrusion. Few studies reported the change in overjet immediately following intrusion. The ratios varied from as low as 1:0.03 [32] to as high as 1:1.1 [25]. Since the overjet is measured at the incisal edge of the lower incisor, different arcs of rotation will be displayed by the incisal edge depending on their pre-treatment position. Moreover, the axial inclination of the lower incisor may change during the autorotation of the mandible as the lower incisors are moved closer to the muscles of the lower lip out of their equilibrium zone. Predicting the change of the overjet in Class II situations, the clinician can decide whether the overjet will be entirely corrected by mandibular autorotation or other treatment procedures such as premolar extractions will be needed.

In this study, the lower lip moved forward at ratio of 1:0.8 to the amount of maxillary molar intrusion. Akan et al. [31] reported negligible effect on the position of the lower lip immediately following intrusion; the measurement, however, was relative to the E-line whose position will change with the autorotation of the mandible. Steinhäuser et al. [16] found that the lower lip moved forward 23.4% of the mean distance of maxillary impaction. Differences in the response of the soft tissue have been attributed to many factors including initial lip length, thickness, and pre-treatment labial tension [41, 42].

Approaches to predict changes using pre-treatment and post-treatment results varied from using mean ratios, linear regressions, and step-wise regressions. Ratios of means are commonly reported in the literature for the prediction of soft tissue to hard tissue changes following treatment. However, regression analyses were shown to offer more accurate predictions [6, 43]. Ratios of means were reported in this study to facilitate comparison with the published literature. In the present study, the strongest correlation coefficients were reported for the hard tissue points: pogonion (*r* = − 0.88, *P* ≤ 0.001) and menton (*r* = 0.91, *P* ≤ 0.001), whereas prediction equations for the change in soft tissue landmarks were weaker particularly for soft tissue pogonion (*r* = − 0.40, *P* ≤ 0.01) and the lower lip (*r* = − 0.51, *P* ≤ 0.01) compared to soft tissue menton (*r* = 0.77, *P* ≤ 0.001). The soft tissue pogonion and the lower lip can be considered highly susceptible to strain and least reproducible in serial radiographs [42]. Generally, prediction equations for 3-mm molar intrusion yielded results similar to those obtained from the ratios of means obtained in this study.

The primary objective of this paper is to help the clinician predict changes that will happen in key treatment planning parameters when using molar intrusion. The prediction parameters may be used for manual cephalometric predication and to adjust computer software algorithms for visualized treatment outcome prediction. These predictions enable the patient to make informed treatment decisions based on patient satisfaction with the predicted treatment outcome. It is noteworthy that despite the use of cephalometric predictors, patient-centered outcome such as patient satisfaction and patient comfort during treatment are of prime importance and need to be addressed in future studies.

## Conclusions

This study attempted to present mean ratios and regression equations to enable the clinician to predict the change in key treatment parameters with the intrusion of the maxillary posterior teeth.
